# A bacterial isolate from the Black Sea oxidizes sulfide with manganese(IV) oxide

**DOI:** 10.1073/pnas.1906000116

**Published:** 2019-06-03

**Authors:** Jan V. Henkel, Olaf Dellwig, Falk Pollehne, Daniel P. R. Herlemann, Thomas Leipe, Heide N. Schulz-Vogt

**Affiliations:** ^a^Biological Oceanography, Leibniz Institute for Baltic Sea Research Warnemünde, 18119 Rostock, Germany;; ^b^Marine Geology, Leibniz Institute for Baltic Sea Research Warnemünde, 18119 Rostock, Germany;; ^c^Centre for Limnology, Estonian University of Life Sciences, Vehendi Village 61117, Estonia

**Keywords:** manganese reduction, sulfide oxidation, *Sulfurimonas*

## Abstract

Mn is one of the most abundant redox-sensitive metals on earth. Some microorganisms are known to use Mn(IV) oxide (MnO_2_) as electron acceptor for the oxidation of organic compounds or hydrogen (H_2_), but so far the use of sulfide (H_2_S) has been suggested but not proven. Here we report on a bacterial isolate which grows autotrophically and couples the reduction of MnO_2_ to the oxidation of H_2_S or thiosulfate (S_2_O_3_^2−^) for energy generation. The isolate, originating from the Black Sea, is a species within the genus *Sulfurimonas*, which typically occurs with high cell numbers in the vicinity of sulfidic environments [Y. Han, M. Perner, *Front. Microbiol.* 6, 989 (2015)]. H_2_S and S_2_O_3_^2−^ are oxidized completely to sulfate (SO_4_^2−^) without the accumulation of intermediates. In the culture, Mn(IV) reduction proceeds via Mn(III) and finally precipitation of Ca-rich Mn(II) carbonate [Mn(Ca)CO_3_]. In contrast to Mn-reducing bacteria, which use organic electron donors or H_2_, Fe oxides are not observed to support growth, which may either indicate an incomplete gene set or a different pathway for extracellular electron transfer.

In stratified basins, for example the Black Sea, in between the oxygenated surface waters and sulfidic bottom waters a suboxic zone lacking oxygen (O_2_), H_2_S, and mostly also nitrate (NO_3_^−^) has been frequently reported (1). Despite the absence of electron acceptors, high bacterial CO_2_ fixation rates at the border with sulfidic waters were measured, without a known energy metabolism which could fuel growth under these environmental conditions (2, 3). Thermodynamically, a suitable electron acceptor for H_2_S oxidation at this depth would be Mn. Even though Mn concentrations are low (Fig. 1*A*), the oxidized form MnO_2_ is particulate and is therefore transported much faster than dissolved electron acceptors to the sulfidic waters by gravitational sinking (4). Nevertheless, so far all attempts to cultivate microorganisms which oxidize H_2_S with MnO_2_ have failed.

**Fig. 1.**
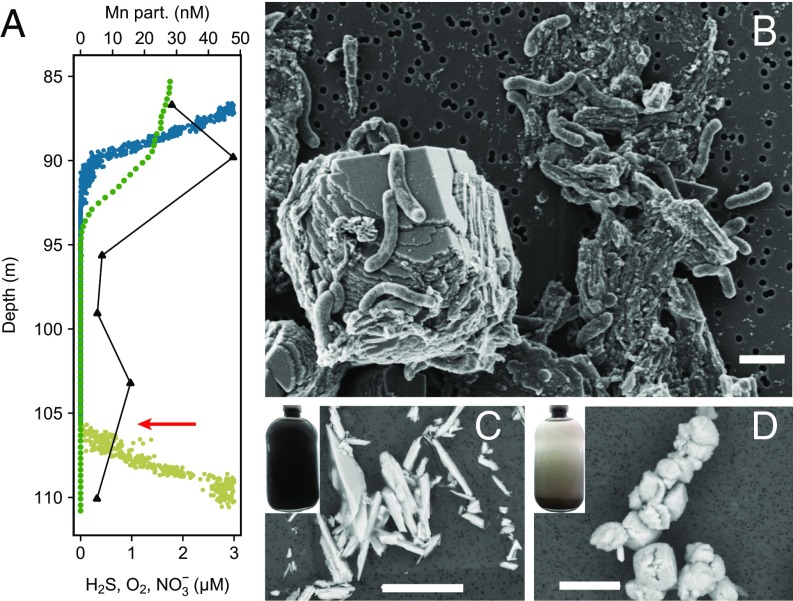
(*A*) Water column profile of the Black Sea suboxic zone: O_2_ (blue), NO_3_^−^ (green), H_2_S (yellow), and particulate (part.) Mn (black triangles). Red arrow indicates sampling for cultivation. Data from ref. 19. (*B*) Scanning electron microscopy (SEM) picture of the isolate ‘*S. marisnigri*.’ (*C* and *D*) Bottle photo and SEM picture of the particulate Mn phase before (*C*, *Insert*) and after (*D*, *Insert*) growth. (Scale bars: *B*, 1 μM; *C* and *D*, 10 μm.)

During an expedition on the research vessel *Maria S. Merian* in November 2013 we sampled the water column of the Black Sea, focusing on the suboxic zone. We took a water sample at the depth of highest abundance of *Epsilonbacteraeota* [12 to 15% (2)] and transferred it into a gas-tight serum bottle containing MnO_2_ (Fig. 1*A*, red arrow). At this depth H_2_S was detectable and neither O_2_ nor NO_3_^−^ was present. After a first enrichment with daily additions of H_2_S resulting in ∼10 to 20 μM concentrations, we transferred a small volume into an artificial medium. From here on we used mainly S_2_O_3_^2−^ for cultivation instead of H_2_S as it is a more convenient electron donor, nontoxic even in higher concentrations, and nonreactive with the MnO_2_ used in our study. A single strain was isolated by repeated series of dilutions-to-extinction transfers.

The isolate belongs to the genus *Sulfurimonas* and its closest cultured relative is *Sulfurimonas gotlandica*, known for the oxidation of H_2_S with NO_3_^−^ in the pelagic redoxcline of the Baltic Sea (5) with a 3% difference in the full 16S rRNA gene sequence. We propose calling the strain ‘*Sulfurimonas marisnigri,*’ in reference to the Latin notation of Pontus Euxı̄nus, meaning *Sulfurimonas* from the Black Sea. The cells are slightly curved, with lengths of 1 to 4 μm and widths of 200 to 300 nm (Fig. 1*B*). ‘*S. marisnigri*’ grows autotrophically with doubling times of 9 to 13 h during the exponential growth phase and reaches a final cell density of 3 to 6 × 10^7^ cells per mL after 7 to 10 d (Fig. 2*A*). Toward the end of the growth phase, the medium turns from black to brownish-gray (Fig. 1 *C* and *D*, *Inserts*), due to the reduction of MnO_2_ and precipitation of Mn(Ca)CO_3_ (Fig. 2*B*). Even though this may be an artifact due to the cultivation conditions, this particular mineral phase was reported in exceptional amounts from the anoxic basins of the Baltic Sea, but the mechanism of its formation is still under debate (6). Cultivation of ‘*S. marisnigri*’ with NO_3_^−^ and successive additions of H_2_S resulted in growth and undetectable H_2_S levels in the culture, indicating a principal ability to use H_2_S directly as electron donor. In contrast to *Shewanella oneidensis* and *Geobacter metallireducens*, attempts to cultivate ‘*S. marisnigri*’ with amorphous FeOOH, goethite (α-FeOOH), Fe_2_O_3_, and FeCl_3_ were unsuccessful, leading to the conclusion that Fe(III) was not a viable electron acceptor under these conditions. This may be due to the absence of a critical protein component for the reduction of Fe oxides or could indicate that extracellular electron transfer onto MnO_2_ might function in a different manner.

**Fig. 2.**
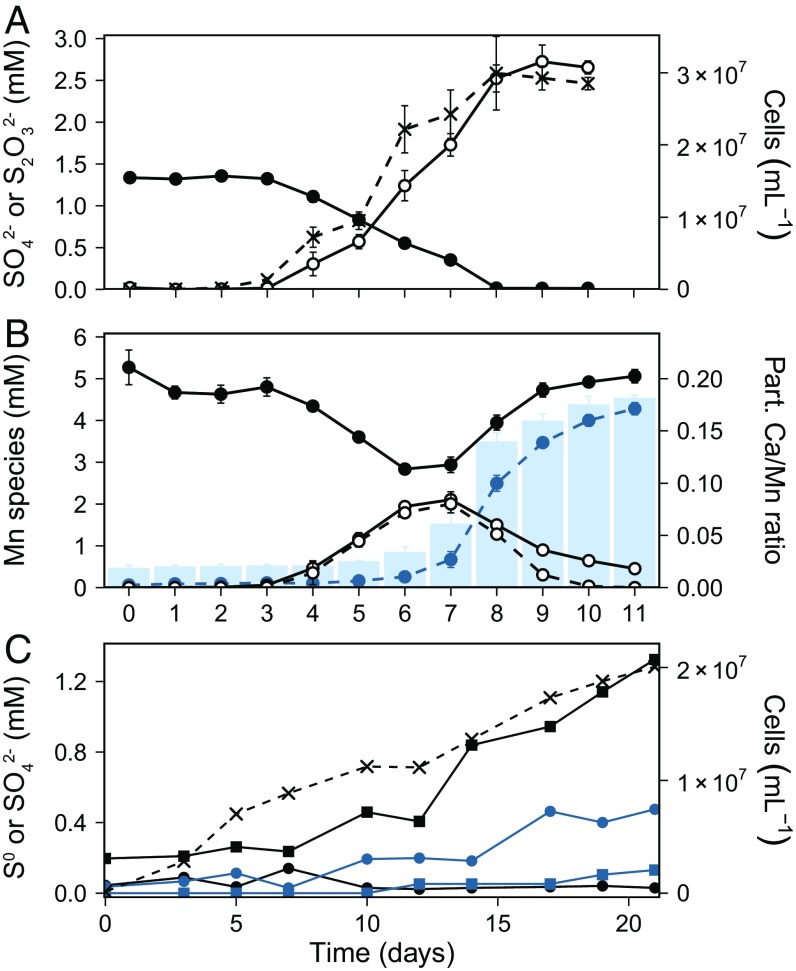
Growth of ‘*S. marisnigri*’ with MnO_2_ and S_2_O_3_^2−^ (*A* and *B*) and MnO_2_ and H_2_S (*C*). (*A*) Increasing cell counts (crosses, dashed line) concurrent with the complete oxidation of S_2_O_3_^2−^ (black solid circles) to SO_4_^2−^ (black open circles). (*B*) Occurrence of different Mn species during growth: particulate (part.) Mn [MnO_2_ and later Mn(Ca)CO_3_, black solid circles], dissolved (diss.) Mn (black open circles, solid line), and diss. reactive Mn (Mn^3+^, black open circles, dashed line). The reduction of part. MnO_2_ results in an equal increase of diss. Mn, almost exclusively Mn^3+^, until day 6. Molar ratio of Ca to Mn in the particulate phase (bars) indicates the precipitation of Mn(Ca)CO_3_ which was used to calculate the amount of reduced Mn in the particulate phase (blue solid circles, dashed line). (*C*) Cultivation of ‘*S. marisnigri*’ with MnO_2_ and constant addition of 3 μL⋅min^−1^ (until day 12) and after that 7.5 μL⋅min^−1^ of an ∼10 mM Na_2_S stock solution results in growth (crosses, dashed line), accumulation of SO_4_^2−^ (black squares), and low concentration of S^0^ (black solid circles). In the sterile control (blue), S^0^ (solid blue circles) is accumulating and concentrations of SO_4_^2−^ (blue squares) increase just slightly.

Growth of ‘*S. marisnigri*’ with MnO_2_ and a constant supply of H_2_S resulted in accumulation of SO_4_^2−^ as cell numbers increased, and negligible concentrations of elemental sulfur (S^0^). In the sterile control, however, S^0^ accumulated and SO_4_^2−^ increased just slightly (Fig. 2*C*). Likewise, in the central gyres of the Black Sea S_2_O_3_^2−^ and sulfite (SO_3_^2−^) were undetectable (7) and S^0^ occurred in nanomolar concentrations (8). Growth with MnO_2_ and S_2_O_3_^2−^ was accompanied by the complete oxidation of S_2_O_3_^2−^ to SO_4_^2−^ with a stoichiometry of 1:2 (Fig. 2*A*) and following Eq. **1**:

$$4\;MnO_{2}\;+\;S_{2}{O_{3}}^{2-}\;+\;6\;H^{+}\;\to \;4\;Mn^{2+}\;+\;2\;S{O_{4}}^{2-}\;+\;3\;H_{2}O.$$ [1]

As with H_2_S, no detectable accumulation of S^0^ and SO_3_^2−^ was observed with S_2_O_3_^2−^ as electron donor. Growth was observed concurrent with the reduction of Mn(IV) to Mn(III) from day 3 to 6 and continued with the reduction of Mn(III) to Mn(II), leading to the precipitation of Ca-rich particles (Fig. 2 *A* and *B*), in contrast to *S. oneidensis*, where growth is only supported by the reduction of Mn(III) to Mn(II) (9). Total inorganic carbon (TIC) analysis of the particulate fraction at the end of the growth phase confirmed the formation of carbonate. The Ca-to-Mn ratio of single Mn(Ca)CO_3_ precipitates was determined using SEM and energy dispersive X-ray microanalysis (EDX). The mean ratio of 0.19 with an SD of 0.03 in cube-shaped precipitates was used to estimate the proportion of reduced Mn in the particulate phase (Fig. 2*B*, blue dashed line). With this approach, we can show that MnO_2_ was almost completely reduced and transformed to Mn(Ca)CO_3_.

We excluded disproportionation of S_2_O_3_^2−^as an alternate explanation for our findings. Cultivation of ‘*S. marisnigri*’ solely on S_2_O_3_^2−^ as an energy source did not result in growth and formation of H_2_S or SO_4_^2−^. This is supported by cultivation of ‘*S. marisnigri*’ with MnO_2_ and a surplus of S_2_O_3_^2−^, in which the concentration of S_2_O_3_^2−^ remained constant after the depletion of MnO_2_. Finally, disproportionation in the presence of Fe oxides would have led to the precipitation of FeS and FeS_2_, which was not the case.

The isolate belongs to the group of *Epsilonbacteraeota* which is reported to be highly abundant in the redox trasition zones of, for example, the Black Sea (12% of the total bacterial community), the Baltic Sea (21%), and the Cariaco Basin (27%) (2, 10). In these systems, *Epsilonbacteraeota* can be responsible for up to 100% of the dark CO_2_ fixation activity in the absence of O_2_ and often NO_3_^−^. Therefore, Jost et al. (3) and Taylor et al. (11) already suggested a potentially MnO_2_-dependent H_2_S oxidation by autotrophic bacteria.

In addition to its presence in pelagic environments, the genus *Sulfurimonas* is globally abundant in redox transition environments such as hydrothermal vents and marine sediments (12). In sediments, the addition of MnO_2_ is thought to promote the production of SO_4_^2−^, apparently depending on microbial activity and leading to the precipitation of Mn(Ca)CO_3_ (13). In those experiments, addition of FeOOH did not stimulate SO_4_^2−^ production. Those findings fit remarkably well to our observations in pure culture, suggesting that an organism with a physiology similar to that of ‘*S. marisnigri*’ may have been responsible for the observed activity.

A further indication that bacterial H_2_S oxidation with MnO_2_ may be of more general importance is the formation of intermediate Mn^3+^ in our cultures, which we detected indirectly as dissolved reactive Mn (14) (Fig. 2*B*). Mn^3+^ was reported to be a major constituent of the marine Mn cycle both in sediments (15) and in the water column of the Black Sea (16) in the absence of O_2_ and H_2_S (17). So far, known processes mediating Mn^3+^ formation are the oxidation of organic matter with MnO_2_ reduction, enzymatic oxidation of Mn^2+^, and the abiotic reaction of MnO_2_ with Fe(II) and H_2_S (15, 18). Our study adds another biologically mediated process via lithotrophic MnO_2_ reduction, which can promote the buildup of Mn^3+^, as observed both in marine sediments and across pelagic redoxclines. In conclusion, we suggest that this bacterial metabolism, which we prove here in pure culture, may be widespread in pelagic redoxclines and to a minor extent in marine sediments where H_2_S is produced and Mn is present in sufficient amounts with important consequences for Mn and S cycling.

## Materials and Methods

### Gases and Nutrients.

Gases and nutrients in the water column were measured as reported in Schulz-Vogt et al. (19).

### Cultivation.

Water samples were taken at 44°−16.7586 N and 36°−18.9567 E at the depth indicated in Fig. 1. Culture purity was ensured by sequencing, lack of growth in organic-rich media, and by microscopy. Medium for cultivation and experiments was prepared anaerobically following the technique described by Widdel and Pfennig (20) based on a SO_4_^2−^-free artificial seawater with a salinity of 21 and a pH of 7.5. The following sterile and anoxic components were added (milliliters per liter): 1 M NaHCO_3_, 30; 1 mM NH_4_Cl, 20; Pfennig’s trace elements solution SL7, 1; 10 mM Na_2_HPO_4_, 1; and vitamin solution, 0.42. Vitamin solution contained (milligrams per milliliter) the following: B_12_, 0.1; inositol, 0.1; biotin, 0.1; folic acid, 0.1; PABA, 1; nicotinic acid, 10; d-pantothenate, 10; and thiamine, 20. Technical MnO_2_ provided as electron acceptor was purchased from Merck and additionally grinded with an agate ball mill.

### Molecular Analysis and Bioinformatics.

DNA was extracted using QIAamp DNA Mini Kit (51306) following the manual. For PCR we used Thermo Fisher Scientific Kit EP0072 according to the manufacturer’s protocol description and primers 27f -1492r. PCR products were purified with the Agentcourt AMpure XP (Beckman Coulter GmbH) magnetic beads and cloned into the vector pSC-A-amp/kan (StrataClone PCR Cloning Kit, competent cells Strataclone SoloPack) following the manual. Clones were sequenced (LGC) forward and reverse using vector primers, trimmed, manually corrected, assembled and deposited at NCBI GenBank (accession nos. MF563385–MF563475).

### Cell Counting.

A glutaraldehyde-fixed sample (2.5% final concentration) was treated with at least five times the volume of a hydroxylamine solution (1.5 M NH_2_OH⋅HCl dissolved in 0.25 M HCl) to dissolve Mn particles. The mixture was treated with ultrasonic for 5 min and subsequently filtered onto a 0.2-μm polycarbonate filter and embedded in DAPI-containing oil. Cells were enumerated with an epifluorescence microscope at 1,000× magnification.

### S_2_O_3_^2−^ and SO_3_^2−^.

We added 0.5 mL 0.2-μm-filtered subsample to 25 μL of Hepes/EDTA buffer (200 mM/50 mM in MilliQ) and 25 μL of the monobrombimane solution (48 mM in acetonitrile) and incubated at room temperature for 30 min in the dark. Derivatization was stopped by adding 25 μL of methanesulfonic acid (324 mM in MilliQ water). Samples were diluted with MilliQ water and measured daily. Samples and calibration series were analyzed using a BioTek HPLC System with pump 525 (1 mL⋅min^−1^), oven 582 (40 °C), column LiChrospher 60 RP-Select B (5 μm) 125 × 4, and a Jasco FP 1520 detector (excitation at 380 nm, emission at 480 nm). Data were analyzed with the program Geminyx III Version 1.10.3.7. Eluent A contained 0.25% acetic acid and eluent B 100% methanol. The gradient protocol was as follows: 0 min 100% A, 2 min 100% A, 5.5 min 92% A, 8 min 68% A, 12 min 68% A, 13 min 0% A, 18 min 0% A, 19 min 100% A, and 23 min 100% A. With these adjustments, S_2_O_3_^2−^ peak appeared after 10 min and SO_3_^2−^ peak after 8 min.

### S^0^.

A 900-μL sample was added to 100 μL of zinc acetate solution (5% wt/vol). Then, 500 μL chloroform were added and intensively mixed for 2 min. One hundred microliters of the chloroform phase were diluted with 400 μL methanol and measured with HPLC using BioTek HPLC System with pump 525 (1 mL⋅min^−1^), oven 582 (25 °C), column LiChrospher 100 RP-18 B (5 μm) 125 × 4, and DAD 545V detector (wavelength 265 nm and 6-nm bandwidth). Data analysis was as described above. An isocratic gradient with 100% methanol was applied. With these adjustments, the peak appeared after 4.4 min.

### Mn and Ca.

Part. Mn and Ca dissolved in hydroxylamine solution as well as total diss. (<0.2 μm) Mn and diss. reactive Mn [dMn_react_, mainly comprising Mn^3+^ (14)] were measured by ICP-OES (iCAP 7400 Duo; Thermo Fisher Scientific) using an external calibration and Sc as internal standard. Precision and accuracy were checked by international reference materials (SGR-1b for the part. and SLEW-3 for the diss. fraction) and were below 2%.

### SEM-EDX.

A Zeiss Merlin Compact SEM (variable pressure, in-lens SE and BSE detector) equipped with EDX (Oxford Instruments) was used to identify Mn(Ca)CO_3_ precipitates and to directly quantify the Ca-to-Mn ratios. The sample preparation was done as described elsewhere (19). Reduced Mn in the part. phase was calculated with Eq. **2**:

$$part.red.Mn\;=part.\;Mn\;*\;\frac{total\;part.\;Ca/Mn\;by\;ICP-OES}{spot\;Ca/Mn\;by\;SEM-EDX\;in\;Mn\left(Ca\right)CO_{3}}.$$ [2]

### TIC.

Dried material was treated with 40% H_3_PO_4_ and the released CO_2_ was analyzed by an IR detector (multi-EA 4000; Analytic Jena). Pure standard CaCO_3_ (12.0% TIC) was used for calibration.
